# Regulation of Progranulin Expression in Human Microglia and Proteolysis of Progranulin by Matrix Metalloproteinase-12 (MMP-12)

**DOI:** 10.1371/journal.pone.0035115

**Published:** 2012-04-11

**Authors:** Hyeon-Sook Suh, Namjong Choi, Leonid Tarassishin, Sunhee C. Lee

**Affiliations:** Department of Pathology, Albert Einstein College of Medicine, Bronx, New York, United States of America; University of North Dakota, United States of America

## Abstract

**Background:**

The essential role of progranulin (PGRN) as a neurotrophic factor has been demonstrated by the discovery that haploinsufficiency due to *GRN* gene mutations causes frontotemporal lobar dementia. In addition to neurons, microglia *in vivo* express PGRN, but little is known about the regulation of PGRN expression by microglia.

**Goal:**

In the current study, we examined the regulation of expression and function of PGRN, its proteolytic enzyme macrophage elastase (MMP-12), as well as the inhibitor of PGRN proteolysis, secretory leukocyte protease inhibitor (SLPI), in human CNS cells.

**Methods:**

Cultures of primary human microglia and astrocytes were stimulated with the TLR ligands (LPS or poly IC), Th1 cytokines (IL-1/IFNγ), or Th2 cytokines (IL-4, IL-13). Results were analyzed by Q-PCR, immunoblotting or ELISA. The roles of MMP-12 and SLPI in PGRN cleavage were also examined.

**Results:**

Unstimulated microglia produced nanogram levels of PGRN, and PGRN release from microglia was suppressed by the TLR ligands or IL-1/IFNγ, but increased by IL-4 or IL-13. Unexpectedly, while astrocytes stimulated with proinflammatory factors released large amounts of SLPI, none were detected in microglial cultures. We also identified MMP-12 as a PGRN proteolytic enzyme, and SLPI as an inhibitor of MMP-12-induced PGRN proteolysis. Experiments employing PGRN siRNA demonstrated that microglial PGRN was involved in the cytokine and chemokine production following TLR3/4 activation, with its effect on TNFα being the most conspicuous.

**Conclusions:**

Our study is the first detailed examination of PGRN in human microglia. Our results establish microglia as a significant source of PGRN, and MMP-12 and SLPI as modulators of PGRN proteolysis. Negative and positive regulation of microglial PGRN release by the proinflammatory/Th1 and the Th2 stimuli, respectively, suggests a fundamentally different aspect of PGRN regulation compared to other known microglial activation products. Microglial PGRN appears to function as an endogenous modulator of innate immune responses.

## Introduction

Progranulin (PGRN) is a growth factor widely expressed in mammalian tissues with highest levels in epithelial and myeloid cells [1]–[3], where it is involved in cell proliferation, wound healing and modulation of inflammation [4], [5]. PGRN contains seven and half granulin domains connected by linker regions. Proteolytic cleavage of PGRN by neutrophil elastase or proteinase 3 generates ∼6 kDa granulins and other molecular weight peptides [1], [2], [5]–[8]. This process can be inhibited by secretory leukocyte protease inhibitor (SLPI) [8]. The full-length PGRN and granulin peptides have been shown to have opposite roles in inflammation [7], [9]. For example, during wound healing, PGRN inhibits neutrophil activation by TNFα but granulins promote epithelial production of neutrophil chemoattractant IL-8 [8].

PGRN has gained much attention with the discovery that haploinsufficiency resulting from the *GRN* gene mutations can cause frontotemporal lobar degeneration (FTLD) [10]–[12], indicating that adequate expression of PGRN is essential for normal CNS aging. PGRN is expressed primarily by neurons and microglia in the CNS [2]. Increased microglial PGRN immunoreactivity is reported in several human CNS diseases including Alzheimer's disease, multiple sclerosis, FTLD, and HIV encephalitis [10], [13], [14] (HS and SCL, unpublished). A detailed analysis of PGRN mRNA in FTLD brains showed an overall increase indicating that PGRN transcription from the normal allele can be upregulated and that PGRN might be separately regulated in neurons and microglia [15], [16]. In addition, PGRN is also dysregulated in the periphery in patients with CNS diseases. For example, peripheral blood PGRN mRNA levels are increased in AD patients [17], whereas plasma PGRN protein levels are reportedly decreased in children with autism [18]. The cellular origins and the molecular mechanisms behind PGRN dysregulation in these patient populations are largely unexplored.

To model FTLD caused by genetic PGRN deficiency, gene knockout (*Grn*−/−) mice have been generated and characterized. While these studies support the general idea that PGRN contributes to normal aging, the CNS abnormalities in these mice are quite subtle [19]–[21]. In addition to increased gliosis and senescence, *Grn*−/− mice also show exaggerated inflammation and impaired host defense. Specifically, LPS-challenged *Grn*−/− macrophages reportedly produce higher amounts TNFα and IL-6 but less IL-10 [20]. *Grn*−/− mice also show defective bacterial clearance from the brain [20]. Other somewhat conflicting results are also reported, including PGRN being a necessary cofactor for TNFα and IL-6 production in mouse macrophages challenged with the TLR9 ligand [22]. In human macrophages, Okura et al., reported that PGRN *augmented* TNFα and IL-1β expression [23]. Other aspects of macrophage biology that have been shown to be affected by PGRN include phagocytosis [2], [24], migration [4], [25], and TNFα signaling [26]. PGRN has also been implicated in the regulation of phagocytosis of apoptotic neurons (programmed cell death) during *C. elegans* development [27].

Despite the importance of microglial PGRN in the CNS, little information is available regarding the regulation of expression and function of PGRN in microglia. In the current study, we examined PGRN production and proteolytic cleavage in primary cultures of human microglia, as well as its role in LPS- and poly IC-induced cytokine production. The results of this study are consistent with the idea that microglial PGRN is a necessary factor for the CNS innate immune responses.

## Results

### Expression of PGRN by cultures of primary human microglia (Figure 1)

**Figure 1:**
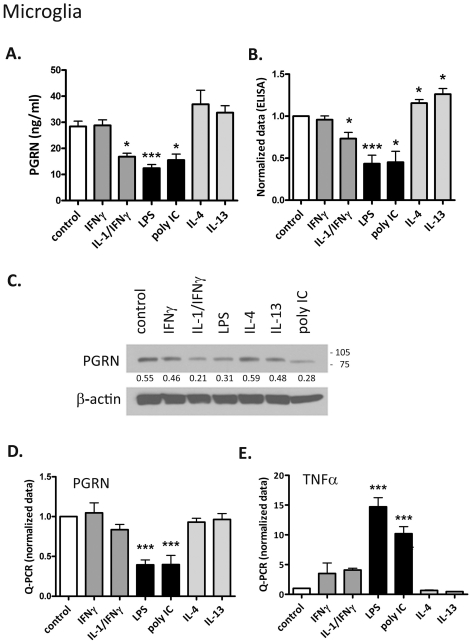
Proinflammatory stimuli suppress microglial PGRN expression. Microglial cultures were treated with IFNγ ± IL-1 (10 ng/ml each), LPS (100 ng/ml), poly IC (10 µg/ml), IL-4 (10 ng/ml), IL-13 (10 ng/ml) or medium alone (control). PGRN expression was examined by ELISA (A & B), western blot (C) or Q-PCR (D &E). Representative ELISA data from a single microglial case are shown in (A), and pooled data from 3–8 different cases are shown in (B) (all 24 h stimulation). All samples were tested in triplicates. (C) Representative western blot (24 h stimulation). Densitometric ratios to β-actin are shown below the blot. (D) Pooled normalized Q-PCR data (6 h stimulation) for PGRN from 3 different cases are shown. (E) TNFα mRNA data are shown as a control. Data are mean ± SD. One-way ANOVA with Dunnett's multiple comparison tests was performed for (A). For all others (normalized data), one sample t-test was performed. * p<0.05, ** p<0.01, ***p<0.001. The results show that microglial PGRN is suppressed by proinflammatory stimuli.

Microglial cultures were treated with different types of inflammatory stimuli: Th1 cytokine (IFNγ), Th2 cytokines (IL-4 or IL-13), TLR ligands (poly IC or LPS) or pro-inflammatory cytokine (IL-1β). PGRN expression was examined by ELISA, western blot and Q-PCR. ELISA data from a representative microglial case and normalized pooled data from multiple microglial cases (all 24 h stimulation) are shown in Figure 1A and B, respectively. Microglia constitutively secreted nanogram levels of PGRN (also see below), which were suppressed by LPS, poly IC or IL-1/IFNγ or increased by IL-4 or IL-13. The most consistent change was suppression of PGRN secretion by LPS (∼50% reduction, p<0.001). A representative western blot (intracellular PGRN) is shown in Figure 1C (24 h post-stimulation). A dominant band corresponding to ∼90 kDa, consistent with reported glycosylated PGRN [1], [2] was detected in all microglial samples. The results show a trend similar to ELISA data, i.e., suppression by proinflammatory stimuli (IL-1/IFNγ, LPS and poly IC), but preservation by the Th2 cytokines (IL-4 or IL-13). Figure 1D is a pooled normalized Q-PCR data (6 h stimulation) showing significant inhibition by LPS and poly IC. TNFα mRNA was determined as an internal control for cytokine/TLR ligand activity ( Figure 1E ). Together, these results in microglia show that proinflammatory stimuli (particularly the TLR3/4 ligands) suppress PGRN expression, and that the Th2 cytokines either preserve or even elevate secreted PGRN levels (see Discussion).

### Expression of PGRN by human fetal astrocytes (Figure 2)

**Figure 2:**
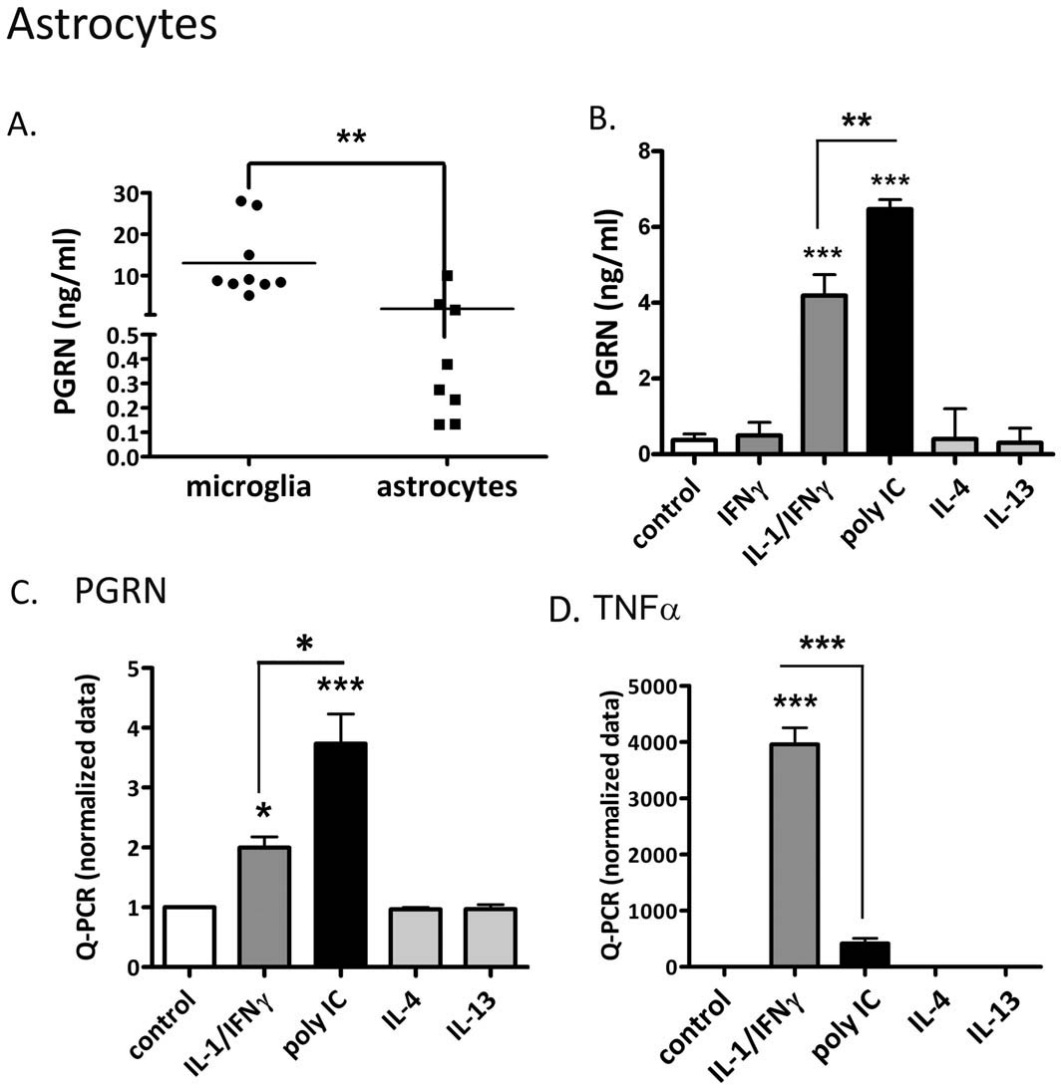
Proinflammatory stimuli enhance astrocyte PGRN Expression. Astrocyte cultures were treated with IFNγ ± IL-1, poly IC, IL-4, IL-13 or medium alone (control) and PGRN expression was examined by ELISA and Q-PCR. (A) Secreted PGRN levels in control microglia and astrocyte cultures determined by ELISA (24 h) show that microglia produce larger amounts of PGRN than astrocytes. Each symbol represents a different case. (B) Representative ELISA data from cytokine-stimulated astrocyte cultures (24 h stimulation). (C, D) Pooled normalized Q-PCR data for PGRN and TNFα from astrocyte cultures (6 h stimulation, n = 5) are shown. Data are mean ± SD. * p<0.05, ** p<0.01, *** p<0.001. The results show that proinflammatory stimuli induce astrocyte PGRN production.

Astrocyte cultures were also examined for PGRN expression, essentially as described for microglia, except LPS was omitted because human astrocytes are non-responsive to LPS. The amounts of secreted PGRN in untreated astrocyte cultures were significantly lower than those in microglial cultures (average 1.98 *versus* 13.04 ng/ml), with many cases showing pg/ml levels (Figure 2A). Interestingly, however, astrocyte PGRN showed a response opposite of microglia, i.e., enhancement by proinflammatory stimuli (IL-1/IFNγ or poly IC). Results of ELISA assay are shown in Figure 2B. Pooled Q-PCR data are shown in Figure 2C. TNFα mRNA expression was shown as a control (Figure 2D). For astrocyte PGRN production, poly IC appeared to be more potent stimulus than IL-1/IFNγ, whereas for astrocyte TNFα, IL-1/IFNγ was a much stronger stimulus than poly IC.

### PGRN is cleaved by macrophage elastase MMP-12 in human microglia (Figure 3)

**Figure 3:**
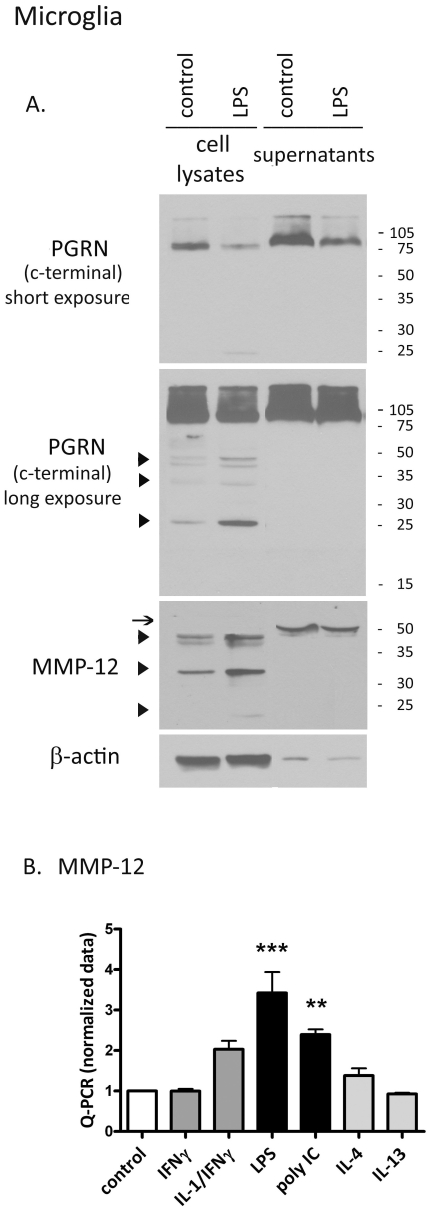
PGRN is cleaved by macrophage elastase MMP-12 in human microglia. (**A**) Microglia were incubated with medium alone (control) or LPS for 24 h. Culture supernatants were concentrated ∼25 fold using a 3 kDa cutoff filter. Equal amounts (30 µg) of protein from cell lysates and culture supernatants were loaded in each lane. Blots were probed for PGRN using a C-terminal specific antibody, and for MMP-12 and β-actin. Data are representative of three independent experiments with similar results. (B) Microglia cultures were examined for MMP-12 mRNA expression by Q-PCR (6 h stimulation). Pooled normalized data from 3 different cases are shown. ** p<0.01, ***p<0.001.

In the periphery, PGRN has been shown to be cleaved to smaller granulin peptides by proteolytic enzymes such as neutrophil elastase or proteinase-3 [7], [8]. PGRN cleavage by microglial enzymes has not been examined. We therefore tested whether macrophage elastase MMP-12 could degrade PGRN. We first examined microglial culture (cell lysates and cell supernatants) for PGRN degradation products by immunoblotting with an antibody against C-terminal PGRN (Figure 3A). Culture supernatants were concentrated using a centrifugal filter device with a 3 kDa molecular weight cutoff. The blots were probed for PGRN, MMP-12 and β-actin. Figure 3A PGRN blot with short exposure (top panel) shows results similar to those shown in Figure 1B, with a predominant band consistent with ∼90 kDa [2]. The secreted PGRN had slightly higher molecular mass, possibly indicating additional protein modifications prior to secretion. Upon long exposure (middle panel), multiple PGRN cleavage products were apparent (∼45 kDa, 42 kDa, 35 kDa and 25 kDa) in cell lysates but not in culture supernatants. Importantly, while LPS reduced the amount of PGRN in both cell lysates and supernatants (top panel), it increased the amount of PGRN cleavage products (middle panel).

The enzyme responsible for PGRN cleavage in microglia or macrophages is not known. We tested a candidate enzyme MMP-12 (macrophage elastase) in our culture. Western blot analysis showed that MMP-12 protein is present in both control and LPS-treated microglia in intracellular and extracellular compartments (Figure 3A, bottom panel). The proenzyme for MMP-12 is ∼54 kDa, and is rapidly activated to a 45 kDa form, and later to a 22 kDa form, and microglial cell lysates show all three bands, as well as an additional ∼35 kDa band [28]–[30], while the secreted MMP-12 was consistent with the MMP-12 proenzyme (54 kDa). Figure 3B shows Q-PCR analysis of microglial MMP-12, which showed that both LPS and poly IC significantly increased the amount of MMP-12 mRNA. Together, these results show that in microglia MMP-12 and PGRN cleavage (activation) occurs mostly intracellularly (see Discussion), consistent with the idea that microglial PGRN is cleaved by MMP-12. LPS increases PGRN cleavage, while reduces the amount of total PGRN.

### PGRN interacts with MMP-12 in microglia (Figure 4)

**Figure 4:**
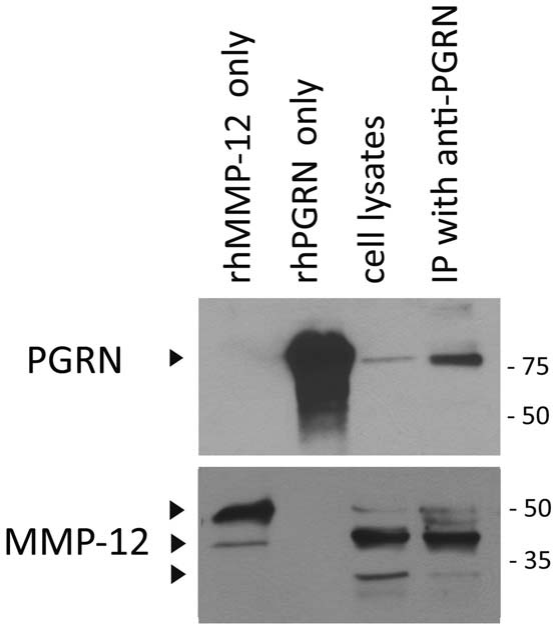
PGRN interacts with MMP-12 in microglia. Microglial cell lysates (untreated, control culture) were immunoprecipitated (IP) with anti-PGRN antibody and immunoblotted with anti-PGRN or anti-MMP-12 antibody. Recombinant MMP-12, PGRN, and non-IP microglial cell lysates were also analyzed in parallel. Microglial cell lysates show ∼90 kDa PGRN and ∼54, 45 and 22 kDa MMP-12 bands (arrowheads). Following IP with anti-PGRN, the ∼45 kDa MMP-12 band is prominent, indicating that PGRN and active MMP-12 interact with each other in microglia.

To determine the possible molecular interaction between PGRN with MMP-12 directly, we performed immunoprecipitation (IP) assay of untreated (control) microglial culture lysates. Cell lysates were immunoprecipitated with a rabbit C-terminal PGRN antibody, then immunoblotted for PGRN and MMP-12. To avoid the interference from denatured IgG, clean-blot IP detection reagent was used for detection of the target antigens. Microglial cell lysates showed ∼90 kDa PGRN and ∼54, 45 and 22 kDa MMP-12 bands consistent with the data in Figure 3A (lane 1). Following IP with anti-PGRN, all three MMP-12 bands were detected, with the ∼45 kDa band being the most prominent. These results show that PGRN and (active) MMP-12 physically interact in microglia and further strengthen the idea that MMP-12 cleaves PGRN.

### MMP-12 cleaves recombinant PGRN (Figure 5)

**Figure 5:**
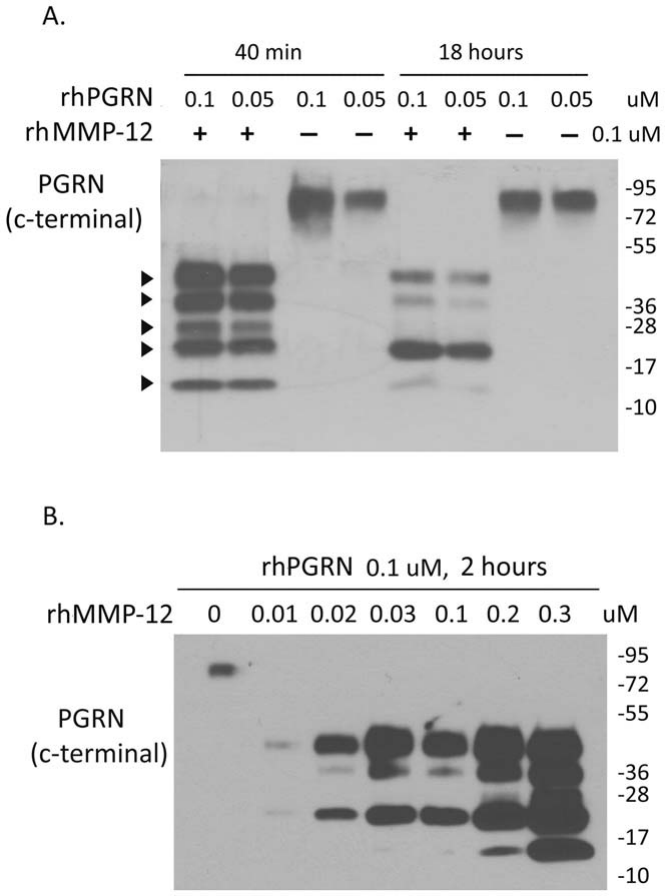
MMP-12 cleaves recombinant PGRN. (A) Varying concentrations of recombinant PGRN (0.05 or 0.1 µM) were incubated with or without activated recombinant MMP-12 (0.1 µM) in a specified assay buffer at 37°C for 40 min or 18 h. Samples were fractionated using a 4–15% gradient gel. (B) MMP-12 dose response: PGRN was incubated with increasing concentrations (0.01–0.3 µM) of MMP-12 for 2 h, then separated using a 4–15% gradient gel. In both samples, five different PGRN cleavage products were noted corresponding to ∼45 kDa, 35 kDa, 25 kDa, 19 kDa and 12 kDa (arrowheads).

We next examined whether MMP-12 can cleave PGRN in a cell-free system. Recombinant human (rh) PGRN was incubated with activated rhMMP-12 in a specified assay buffer for 40 min or 18 h at 37°C. The samples were fractionated in a 4–15% gradient gel. Figure 5A shows that only in the presence of MMP-12, PGRN cleavage occurred. Five major molecular species (∼45 kDa, 35 kDa, 25 kDa, 19 kDa and 12 kDa) of PGRN degradation products were detected at 40 min, with the ∼19 kDa band becoming intense at 18 h. Figure 5B shows MMP-12 dose-dependent (0.01–0.3 µM, 2 h post incubation) degradation of PGRN.

### The effect of SLPI on MMP-12-induced PGRN cleavage (Figure 6)

**Figure 6:**
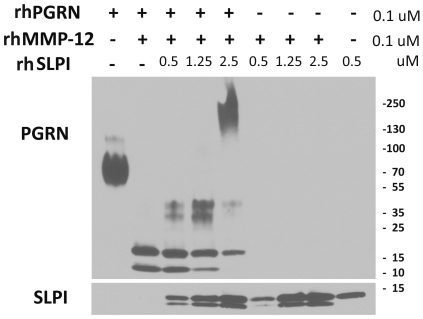
Inhibition of MMP-12-mediated PGRN cleavage by SLPI. Recombinant PGRN (0.1 µM) was incubated with activated MMP-12 (0.1 µM) with or without SLPI at indicated doses (0.5–2.5 µM) for 40 min at 37°C. Western blot was performed for PGRN and SLPI. Results show that MMP-12 mediated PGRN cleavage was dose-dependently inhibited by SLPI. Data are representative of three separate experiments with similar results.

We next examined whether SLPI inhibits MMP-12-induced PGRN cleavage. PGRN was incubated with activated MMP-12 with and without SLPI in a specified assay buffer for 40 min at 37°C, as described in the Methods section. Western blot was performed for PGRN and SLPI. Figure 6 shows that PGRN (0.1 µM) was cleaved only in the presence of MMP-12 (0.1 µM) and this was inhibited by SLPI in a dose-dependent manner (0.5 µM–2.5 µM). At 2.5 µM, SLPI appeared to have induced polymerization of PGRN (∼240 kDa) to prevent degradation. Furthermore, we find that SLPI was cleaved by MMP-12, both in the presence and absence of PGRN.

### SLPI expression by human astrocytes and microglia (Figure 7)

**Figure 7:**
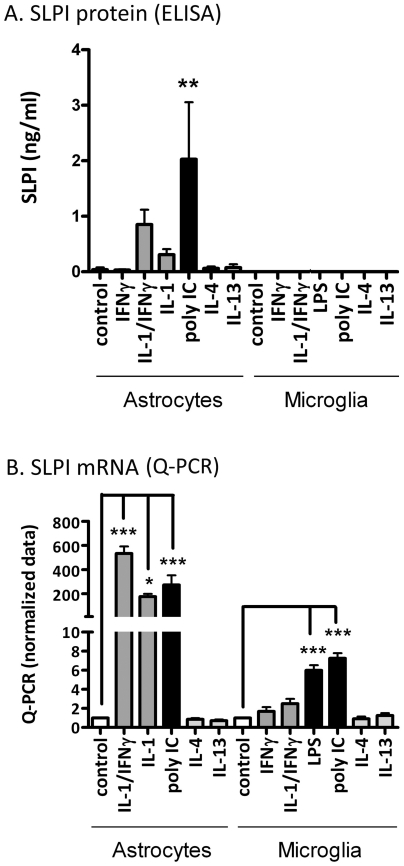
SLPI expression by human astrocytes and microglia. Astrocyte or microglial cultures were treated with IFNγ ± IL-1, LPS, poly IC, IL-4 or IL-13 and SLPI expression was examined by ELISA (24 h) or Q-PCR (6 h stimulation). (A) Representative ELISA data from astrocytes and microglia are shown. Results show that SLPI is produced by activated astrocytes. Results are mean ± SD and are representative of 3–5 separate cases. (B) Pooled normalized Q-PCR data (n = 3) show that SLPI mRNA was induced by proinflammatory stimuli (astrocytes>>microglia). * p<0.05, ** p<0.01, *** p<0.001.

SLPI is a multifunctional protein with many important biological activities but whether SLPI is produced by the cells in the CNS is unknown. Therefore, SLPI expression was determined in astrocyte and microglial cultures. A representative ELISA data (24 h) is shown in Figure 7A. Surprisingly, while astrocytes produced ng/ml levels of SLPI following proinflammatory stimulation, microglial cultures had no detectable SLPI (<25 pg/ml). By Q-PCR (6 h), astrocytes expressed high levels of SLPI mRNA following stimulation with IL-1±IFNγ or poly IC (pooled normalized data) consistent with the ELISA data (Figure 7B). Although SLPI mRNA induction was detectable in microglia following LPS or poly IC stimulation, the levels were ∼100-fold lower than those detected in astrocytes (Figure 7B). These results show that although in rodents, macrophages are the major source of SLPI [31], human microglia (and monocyte-derived macrophages, n = 3, not shown) do not make significant amounts of SLPI. Rather, astrocytes appear to be the main producer of SLPI in human CNS cell cultures.

### Microglial PGRN is an endogenous cofactor for cytokine and chemokine production following TLR3/4 stimulation (Figures 8 and 9)

**Figure 8:**
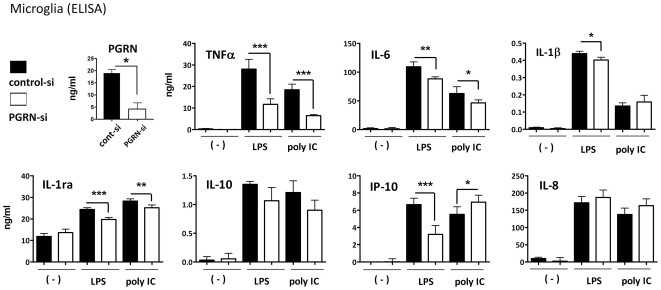
Role of microglial PGRN in TLR3/4-mediated cytokine production. Microglial cultures were treated with siRNA specific for human PGRN (PGRN-si: white symbol) or a control non-targeting siRNA (cont-si: black symbol) for 3 days. Cultures were then stimulated with LPS or poly IC for additional 24 h. (A) PGRN ELISA was performed to determine the effect of PGRN-si in microglia. (B to H) TNFα, IL-6, IL-1β, IL-1ra, IL-10, IP-10 and IL-8 were measured by ELISA in the same culture. Results from a representative experiment are shown. Data are mean ± SD from triplicate samples (* p<0.05, ** p<0.01, ***p<0.001). The results show that PGRN-si reduces the production of multiple cytokines and chemokines induced by LPS or poly IC.

**Figure 9:**
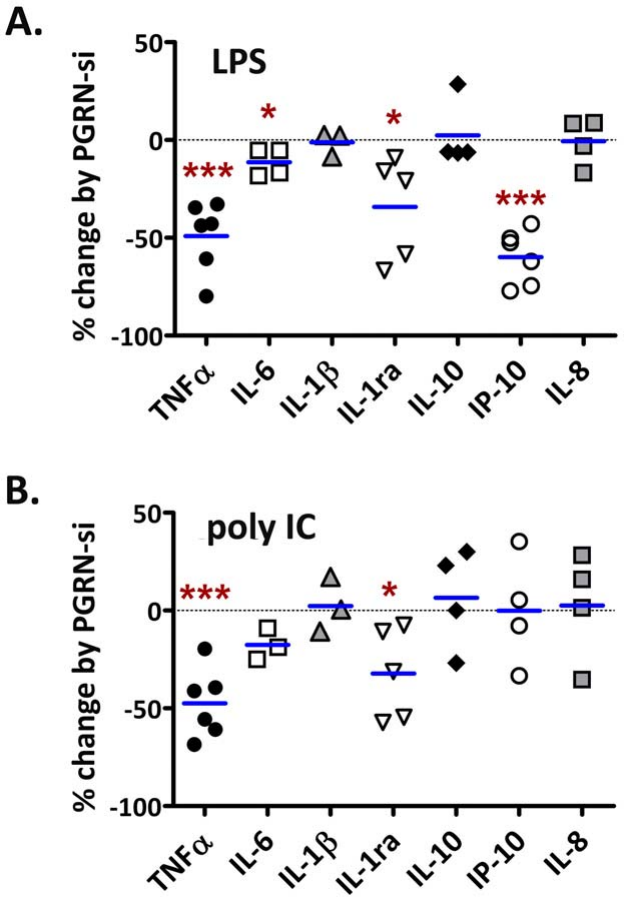
PGRN modulates TLR3/4-mediated cytokine production (pooled data from multiple cases). Microglia were transfected with control or PGRN siRNA for 3 days, then further treated with LPS (A) or poly IC (B) for additional 24 h, then cytokines were measured ELISA as shown in Figure 8. Data are then expressed as % change by PGRN siRNA as calculated by 100×(PGRN siRNA/control siRNA - 1). Zero (dotted line) marks no change. Results shown are from multiple microglial cases with each symbol representing a different case. The data show that PGRN siRNA reduced the amount of microglial TNFα, IL-6, IL-1ra and IP-10 induced by LPS, and TNFα and IL-1ra induced by poly IC. * p<0.05, ** p<0.01, *** p<0.001.

Given the recent evidence that PGRN modulates cytokine production in rodent macrophages [20], [22], we asked whether PGRN modulates microglial cytokine and chemokine production by employing small-interfering RNA (siRNA). Microglial cultures treated with PGRN siRNA (72 h) resulted in an average of ∼80% reduction of PGRN production (Figure 8A). Following treatment with PGRN siRNA (or control non-targeting siRNA), cultures were further exposed to LPS or poly IC for an additional 24 h. ELISA was performed for TNFα, IL-6, IL-1β, IL-1 receptor antagonist (IL-1ra), IP-10, IL-8 and IL-10. Results from a representative microglial case are shown in Figure 8, and pooled data from multiple microglial cases expressed as % change by PGRN siRNA are shown in Figure 9. These results together show that PGRN siRNA reduces the amount of cytokines including TNFα, IP-10, IL-6, IL-1ra, and possibly IL-1β. The degree of inhibition varied depending on the cytokine and the stimulus. The strongest (∼50%) and most significant inhibition was found for TNFα and IP-10. Interestingly, LPS-induced IP-10 but not poly IC-induced IP-10 was inhibited by PGRN siRNA. IL-1ra was also inhibited by ∼25%, and IL-6 was inhibited by ∼10%. PGRN siRNA had no significant effect on IL-1β, IL-8, or IL-10 production (Figure 9). None of the cytokines were significantly increased by PGRN siRNA, indicating that endogenous PGRN had a *stimulatory* role in microglial cytokine production.

## Discussion

To our knowledge, this is the first detailed study of PGRN production in primary CNS cells. Aside from a study of myeloid cell lines reporting increase of PGRN mRNA by cell differentiating agents such as retinoic acid and phorbol ester (PMA) [32], no information is available on the mechanisms that regulate PGRN production from macrophages or CNS cells. Our *in vitro* studies of human fetal microglia and astrocytes show that microglia are the major source of PGRN but that inflammatory stimuli differentially regulate PGRN in the two cell types. Thus, our data supports that microglial PGRN is a constitutively produced neuronal growth factor. Interestingly, the TLR ligands (LPS and poly IC were examined) and the proinflammatory/Th1 cytokines (IL-1/IFNγ) suppressed microglial PGRN, while they increased PGRN production from astrocytes. Human *GRN* promoter contains several consensus sequences involved in cytokine and growth-factor-regulated gene expression, including IL-6 response element, NFL-IL-6 binding sites, and TGFβ-1 inhibitory element [33], [34]. The large number and variety of putative regulatory elements implies considerable complexity and versatility in the regulation of expression of the gene *GRN* [33]. The astrocyte response to IL-1/IFNγ is similar to that of mouse embryonic fibroblasts which showed PGRN induction by TNFα or IL-1β [35], but there is no precedent for the observed suppression of PGRN by inflammatory mediators. While proinflammatory mediators suppressed PGRN, the Th2 cytokines (IL-4 and IL-13) increased PGRN production in microglia, as determined by ELISA. The differential regulation by Th1 vs. Th2 cytokines is in keeping with the reported differential effects of IFNγ (decrease) and IL-4 (increase) in murine microglial IGF-1 [36]. These results suggest the presence of common regulatory elements that control the expression of neuronal growth factors in microglia. The contrasting responses of astrocytes and microglia also indicate that PGRN regulation in monocyte-lineage cells is different from that in epithelial cells and fibroblasts.

Unlike PGRN, its proteolytic products granulins are reported to have proinflammatory activities. The details of granulin production and biological consequences have been studied in neutrophils and epithelial cells [1], [4], [8]. In microglia, we show that PGRN cleavage occurs intracellularly and this is increased by LPS, although LPS suppressed the total amount of PGRN. These results support the scenario that under inflammatory CNS conditions, PGRN production is suppressed while granulin production is increased, switching the role of microglia from a neurotrophic one to an inflammatory one. In order to understand the role of PGRN, we employed PGRN siRNA to silence its expression and this approach proved to be effective in our microglia. Surprisingly, these experiments showed that PGRN positively regulated TLR3/4-induced cytokine and chemokine production. Its effect on TNFα production was most notable. In addition, IP-10, IL-6 and IL-1ra were affected, while IL-1, IL-8 and IL-10 were not affected. This is fitting with the notion that PGRN/granulin contributes to the CNS innate immune responses, rather than inhibiting cytokine production, as has been maintained previously [20].

The profound effect of microglial PGRN in the induction of TNFα shown here probably reflects the complex role of TNFα in the TLR signaling. Following LPS stimulation, initial TNFα production has an important role in early autocrine activation of macrophages [37]. PGRN has recently been found to directly bind to TNFα receptors thereby suppressing TNFα receptor signaling [26]. The role of TNFα in immunity and inflammation is also highly complex. While proven to be harmful in rheumatoid arthritis, it was found to be beneficial in multiple sclerosis patients [38]–[41]. IL-6 also has a complex biological activity. For example, IL-6 induces Th17 differentiation of T cells thereby contributing to autoimmunity, but it also has a neurotrophic activity shared by other gp130 cytokines [42]. IP-10 is a principal chemoattractant for T cells thus is crucial in inflammatory responses, but it has demonstrated detrimental effects on neurons [43]. Our data also suggests that PGRN might contribute to IL-1 antagonism. Therefore, PGRN may help maintain the differential cytokine balance (TNFα, IP-10, IL-6>IL-1) and fine tune the CNS inflammatory response, without compromising the integrity of neural elements.

Our study also establishes PGRN as a MMP-12 substrate. Previously, PGRN has been shown to be cleaved by MMP-14 [44], MMP-9 [45], and ADAMTS7 [46], in addition to neutrophil elastase [8], thus it is likely that multiple enzymes (in addition to MMP-12) can generate granulins in the CNS. Curiously, microglial MMP-12 activation and PGRN cleavage occurred intracellularly but not extracellularly. MMP-12 activity is regulated by several factors including Ca^2+^ concentration and pH [47], thus it is most likely that extracellular PGRN proteolysis will occur under the conditions that sharply increase the Ca^2+^ levels such as during inflammation and cell death [48].

In our study, astrocyte PGRN was increased by IL-1/IFNγ or poly IC, contrary to microglia. Astrocytes contribute considerably less to the overall PGRN pool in the CNS on a per cell basis. Despite our observations, astrocyte contribution to PGRN production *in vivo* might be even smaller, since astrocytes are infrequently observed to be immunoreactive for PGRN in the CNS. The role of astrocytes may be more important as a source of SLPI, an inhibitor of PGRN proteolysis. We show that recombinant SLPI is effective in inhibiting MMP-12-induced PGRN cleavage, though high molar excess (to MMP-12) was required. SLPI at high concentrations caused a shift in PGRN molecular mass, probably reflecting polymerization. In addition, MMP-12 also caused a partial cleavage of SLPI [49], establishing SLPI as another MMP-12 substrate. Therefore, complex interactions involving PGRN, MMP-12 and SLPI are likely to occur *in vivo*.

Another surprising finding of our study is that human microglia produced little or no SLPI. In rodents, macrophages have been shown to be a significant source of SLPI [31], [50], [51]. Few reports exist that demonstrate SLPI production by human macrophages [52]. It is possible that in human microglia (and likely macrophages), SLPI expression is silenced, akin to iNOS expression in human macrophages [53]. In addition to its anti-proteolytic activities, SLPI has other known functions, such as inhibition of HIV [54] and suppression of macrophage activation [31]. For example, extracellular SLPI has been shown to be taken up by macrophages, translocate to the nucleus, and inhibit NF-κB activation and cytokine production [31]. Therefore, astrocyte SLPI might counteract various aspects of microglial activation *in vivo*. Furthermore, the low to absent SLPI expression by human microglia (and macrophages) might render these cells more prone to proinflammatory activation.

Our study sheds light into the PGRN biology in human CNS cells, but also opens up several important questions, such as the molecular mechanisms underlying microglial PGRN gene repression, and the signals that upregulate microglial PGRN expression *in vivo*. It is also unclear whether neuronal PGRN is under similar regulation. The fundamentally different role of microglial (macrophage) PGRN in inflammation (as opposed to PGRN's generally implicated neurotrophic role) might suggest that neuron-autonomous PGRN is important in the maintenance of normal neuronal physiology. The results also suggest that complete absence of PGRN in *Grn−/−* mice may not accurately reflect the relationship between neuronal and microglial PGRN (such as that occurs in FTLD), due to abolition of the reactive PGRN arm. Future studies addressing these issues could aid the understanding of the mechanism by which PGRN deficiency leads to neurodegeneration in FTLD.

## Materials and Methods

### Ethics Statement

Human tissue collection was approved by the Albert Einstein College of Medicine Institutional Review Board (IRB#: 1994-019). Informed written consent was obtained from all participants involved in the study.

### Primary human microglial and astrocyte culture

Human cell cultures were prepared from human fetal abortuses as described with minor modifications [55]. All tissue collection was approved by the Albert Einstein College of Medicine Institutional Review Board. Primary mixed CNS cultures were prepared by enzymatic and mechanical dissociation of the cerebral tissue followed by filtration through nylon meshes of 230- and 130-µ pore sizes. Single cell suspension was plated at 1–10×10^6^ cells per ml in DMEM (Cellgro, Mediatech) supplemented with 10% FBS (Gemini Bio-products, Woodland, CA), penicillin (100 U/ml), streptomycin (100 µg/ml) and fungizone (0.25 µg/ml) (complete medium) for 2–3 weeks, and then microglial cells were collected by aspiration of the culture medium. Monolayers of microglia were prepared in 60-mm tissue culture dishes at 1×10^6^ cells per 5 ml medium or in 96-well tissue culture plates at 3–4×10^4^ per 0.1 ml medium. Four to sixteen hours later, cultures were washed to remove non-adherent cells (neurons and astrocytes). Microglial cultures were highly pure consisting of >98% CD68^+^ cells. Highly enriched human astrocyte cultures were generated by repeated passage of the mixed CNS cultures, as described previously [56]. All cultures were kept as monolayers in DMEM with 5% FCS and antibiotics.

### Cell stimulants and culture treatment

LPS and poly IC were from Sigma-Aldrich (St. Louis, MO), recombinant human (rh) IL-1β, IL-4, IL-13 and IFNγ were from Peprotech (Rocky Hill, NJ). PGRN, MMP-12, and SLPI were from R&D systems (Minneapolis, MN). Culture media were changed to low serum media (DMEM+0.2% FBS) 24 h prior to cell stimulation. All cytokines were used at 10 ng/ml, poly IC at 10 µg/ml, and LPS at 100 ng/ml. MMP-12 was incubated with assay buffer (50 mM Tris, 10 mM CaCl_2_, 150 mM NaCl, 0.05% Brij-35, pH 7.5) at 37°C for 30 h to activate enzyme, following the manufacturer's protocol (R&D Systems).

### Concentration of culture supernatants

Culture supernatants were concentrated ∼25 fold using a centrifugal filter device (Amicon Ultra 3K) from Millipore (Billerica, MA), according to the manufacturer's protocol. Protein concentration was quantified using the Bradford (Bio-Rad) assay.

### Western blot analysis

Western blot analysis was performed as previously described [57] with minor modifications. Briefly, cell cultures in 60 mm dishes were scraped into lysis buffer (Tris-sucrose buffer, pH 7.4) Thirty to fifty micrograms of protein was separated by 12% sodium dodecyl sulfate-polyacrylamide gel. For immunoblotting of recombinant proteins, 4–15% gradient gels (Bio-Rad: Hercules, CA) were used. Proteins were transferred to polyvinylidene difluoride membranes (Millipore Inc.). The membranes were blocked in PBS-0.1% Tween-20 containing 5% nonfat milk and then incubated with antibodies at 4°C for 16 h. Following primary antibodies were used: rabbit polyclonal IgG against C-terminal PGRN (Invitrogen: Camarillo, CA) 1∶100; goat anti-human (full length) PGRN IgG (R&D systems) 1∶1,000; rabbit anti-human MMP-12 (Millipore) 1∶1,000; and goat anti-human SLPI (Life science) 1∶ 250. Secondary antibodies were horseradish peroxidase-conjugated anti-rabbit (Pierce Biotechnology: Rockford, IL) or anti-goat IgG (Southern Biotechnology Associates: Birmingham, AL) at 1∶1,000. Signals were developed using enhanced chemiluminescence (Pierce Biotechnology). All blots were reprobed for β-actin (Sigma) as a protein loading control. Densitometric analysis was performed using the ImageJ software (NIH).

### Enzyme-linked immunosorbent assay (ELISA)

IL-1β, TNFα, IL-6, IL-8, IL-10, IL-1ra and IP-10 were detected using the DuoSets from R&D Systems, as previously described [58]. PGRN levels were determined using either the Human PGRN Quantikine ELISA kit (R&D Systems) or the DuoSet, with similar results. SLPI levels were determined using Human SLPI Quantikine ELISA kit from R&D Systems (sensitivity ∼25 pg/ml). All samples were diluted until the values fell within the linear range of the standard.

### Real-time PCR

Quantitative real-time reverse transcription-PCR (Q-PCR) was performed as described previously [57], [59], using glyceraldehyde 3-phosphate dehydrogenase (GAPDH) and porphobilinogen deaminase (PBDA) as internal control. Following primers were used: PGRN Forward- GAGGACTAACAGGGCAGTGG, Backward- GCCTCTGGGATTGGACAG; SLPI Forward- CCAGTCACTCTGGCACTCAG, Backward- CTGTGGAAGGCTCTGGAAAG; MMP-12 Forward-TGGCCAAGACCTAAGGAATG, Backward- GATGCACATTTCGATGAGGA. Briefly, total RNA was extracted with TRIzol (Invitrogen Life Technologies), and PCR was performed using a SYBR green PCR mix and conducted with ABI Prism 7900HT (Applied Biosystems). The median value of the replicates for each sample was calculated and expressed as the cycle threshold (*C_T_*; cycle number at which each PCR reaches a predetermined fluorescence threshold, set within the linear range of all reactions). Δ*C_T_* was calculated as *C_T_* of endogenous control gene minus *C_T_* of target gene in each sample. The relative amount of target gene expression in each sample was then calculated as 2^Δ*CT*^. Fold change was calculated by dividing the value (2^Δ*CT*^) of test sample by the value (2^Δ*CT*^) of control sample (control = 1).

### Immunoprecipitation

Microglial cell lysates were pre-incubated with protein A agarose beads (Thermo Scientific) for 3 h to reduce non-specific reaction. The supernatants were then mixed with anti-PGRN (C-terminal) for 1 h. The mixtures were then incubated with protein A agarose beads (Thermo Scientific) overnight at 4°C. The beads were boiled in SDS-sample buffer and centrifugated to release the immune complex. The immunoprecipitates were separated in a 12% SDS polyacrylamide gel. The membranes were immunoblotted with anti-rabbit MMP-12 or anti-goat PGRN antibody. For the immunoprecipitated samples, clean-blot IP detection reagent (Thermo Scientific Cat#21230), which detects only specific target antigen without interference from heavy and light chain (denatured IgG), was used instead of secondary antibodies.

### PGRN knockdown by siRNA

Microglia were transfected with 20 nM control non-targeting small-interfering RNA (siRNA) or human PGRN-specific siRNA (Dharmacon, Chicago, IL) with transit-TKO transfection reagents from Mirus (Madison, WI) following the manufacturer's instructions. After incubation with siRNA for 3 to 4 days, cells were washed with fresh medium and then treated with cytokines for an additional 24 h. ELISA was performed to determine the efficiency of PGRN knockdown by siRNA.

### Statistical Analysis

For multiple comparisons, one-way ANOVA with Dunnett's multiple comparison tests was performed. For comparison of two groups, Student's t-test was performed. For comparing normalized data, one sample t-test was used to determine whether the changes were significantly different from control. All data were expressed as mean (± SD). P values<0.05 were considered significant: * denotes p<0.05, ** p<0.01, and *** p<0.001. All statistics were performed using the GraphPad Prism 5.0 software.

## References

[1] He Z, Bateman A (2003). Progranulin (granulin-epithelin precursor, PC-cell-derived growth factor, acrogranin) mediates tissue repair and tumorigenesis.. J Mol Med.

[2] Ahmed Z, Mackenzie IR, Hutton ML, Dickson DW (2007). Progranulin in frontotemporal lobar degeneration and neuroinflammation.. J Neuroinflammation.

[3] Daniel R, Daniels E, He Z, Bateman A (2003). Progranulin (acrogranin/PC cell-derived growth factor/granulin-epithelin precursor) is expressed in the placenta, epidermis, microvasculature, and brain during murine development.. Dev Dyn.

[4] He Z, Ong CH, Halper J, Bateman A (2003). Progranulin is a mediator of the wound response.. Nat Med.

[5] De ML, Van DP (2011). Cellular Effects of Progranulin in Health and Disease.. J Mol Neurosci.

[6] Wang J, Van DP, Cruchaga C, Gitcho MA, Vidal JM (2010). Pathogenic cysteine mutations affect progranulin function and production of mature granulins.. J Neurochem.

[7] Kessenbrock K, Frohlich L, Sixt M, Lammermann T, Pfister H (2008). Proteinase 3 and neutrophil elastase enhance inflammation in mice by inactivating antiinflammatory progranulin.. J Clin Invest.

[8] Zhu J, Nathan C, Jin W, Sim D, Ashcroft GS (2002). Conversion of proepithelin to epithelins: roles of SLPI and elastase in host defense and wound repair.. Cell.

[9] Kojima Y, Ono K, Inoue K, Takagi Y, Kikuta K (2009). Progranulin expression in advanced human atherosclerotic plaque.. Atherosclerosis.

[10] Baker M, Mackenzie IR, Pickering-Brown SM, Gass J, Rademakers R (2006). Mutations in progranulin cause tau-negative frontotemporal dementia linked to chromosome 17.. Nature.

[11] Cruts M, Gijselinck I, van der ZJ, Engelborghs S, Wils H (2006). Null mutations in progranulin cause ubiquitin-positive frontotemporal dementia linked to chromosome 17q21.. Nature.

[12] Sleegers K, Cruts M, Van BC (2010). Molecular pathways of frontotemporal lobar degeneration.. Annu Rev Neurosci.

[13] Pereson S, Wils H, Kleinberger G, McGowan E, Vandewoestyne M (2009). Progranulin expression correlates with dense-core amyloid plaque burden in Alzheimer disease mouse models.. J Pathol.

[14] Vercellino M, Grifoni S, Romagnolo A, Masera S, Mattioda A (2011). Progranulin expression in brain tissue and cerebrospinal fluid levels in multiple sclerosis.. Mult Scler.

[15] Chen-Plotkin AS, Xiao J, Geser F, Martinez-Lage M, Grossman M (2010). Brain progranulin expression in GRN-associated frontotemporal lobar degeneration.. Acta Neuropathol.

[16] Eriksen JL (2010). The enigmatic roles of microglial versus neuronal progranulin in neurological disease.. Acta Neuropathol.

[17] Coppola G, Karydas A, Rademakers R, Wang Q, Baker M (2008). Gene expression study on peripheral blood identifies progranulin mutations.. Ann Neurol.

[18] Al-Ayadhi LY, Mostafa GA (2011). Low plasma progranulin levels in children with autism.. J Neuroinflammation.

[19] Ahmed Z, Sheng H, Xu YF, Lin WL, Innes AE (2010). Accelerated lipofuscinosis and ubiquitination in granulin knockout mice suggest a role for progranulin in successful aging.. Am J Pathol.

[20] Yin F, Banerjee R, Thomas B, Zhou P, Qian L (2010). Exaggerated inflammation, impaired host defense, and neuropathology in progranulin-deficient mice.. J Exp Med.

[21] Yin F, Dumont M, Banerjee R, Ma Y, Li H (2010). Behavioral deficits and progressive neuropathology in progranulin-deficient mice: a mouse model of frontotemporal dementia.. FASEB J.

[22] Park B, Buti L, Lee S, Matsuwaki T, Spooner E (2011). Granulin is a soluble cofactor for toll-like receptor 9 signaling.. Immunity.

[23] Okura H, Yamashita S, Ohama T, Saga A, Yamamoto-Kakuta A (2010). HDL/apolipoprotein A-I binds to macrophage-derived progranulin and suppresses its conversion into proinflammatory granulins.. J Atheroscler Thromb.

[24] Bateman A, Bennett HP (2009). The granulin gene family: from cancer to dementia.. Bioessays.

[25] Eriksen JL, Mackenzie IR (2008). Progranulin: normal function and role in neurodegeneration.. J Neurochem.

[26] Tang W, Lu Y, Tian QY, Zhang Y, Guo FJ (2011). The growth factor progranulin binds to TNF receptors and is therapeutic against inflammatory arthritis in mice.. Science.

[27] Kao AW, Eisenhut RJ, Martens LH, Nakamura A, Huang A (2011). A neurodegenerative disease mutation that accelerates the clearance of apoptotic cells.. Proc Natl Acad Sci U S A.

[28] Dasilva AG, Yong VW (2008). Expression and regulation of matrix metalloproteinase-12 in experimental autoimmune encephalomyelitis and by bone marrow derived macrophages in vitro.. J Neuroimmunol.

[29] Nar H, Werle K, Bauer MM, Dollinger H, Jung B (2001). Crystal structure of human macrophage elastase (MMP-12) in complex with a hydroxamic acid inhibitor.. J Mol Biol.

[30] Arikan MC, Shapiro SD, Mariani TJ (2005). Induction of macrophage elastase (MMP-12) gene expression by statins.. J Cell Physiol.

[31] Jin FY, Nathan C, Radzioch D, Ding A (1997). Secretory leukocyte protease inhibitor: a macrophage product induced by and antagonistic to bacterial lipopolysaccharide.. Cell.

[32] Ong CH, He Z, Kriazhev L, Shan X, Palfree RG (2006). Regulation of progranulin expression in myeloid cells.. Am J Physiol Regul Integr Comp Physiol.

[33] Bhandari V, Daniel R, Lim PS, Bateman A (1996). Structural and functional analysis of a promoter of the human granulin/epithelin gene.. Biochem J.

[34] Frampton G, Invernizzi P, Bernuzzi F, Pae HY, Quinn M (2011). Interleukin-6-driven progranulin expression increases cholangiocarcinoma growth by an Akt-dependent mechanism.. Gut.

[35] Li X, Massa PE, Hanidu A, Peet GW, Aro P (2002). IKKalpha, IKKbeta, and NEMO/IKKgamma are each required for the NF-kappa B-mediated inflammatory response program.. J Biol Chem.

[36] Butovsky O, Landa G, Kunis G, Ziv Y, Avidan H (2006). Induction and blockage of oligodendrogenesis by differently activated microglia in an animal model of multiple sclerosis.. J Clin Invest.

[37] O'Connell RM, Taganov KD, Boldin MP, Cheng G, Baltimore D (2007). MicroRNA-155 is induced during the macrophage inflammatory response.. Proc Natl Acad Sci U S A.

[38] Mohan N, Edwards ET, Cupps TR, Oliverio PJ, Sandberg G (2001). Demyelination occurring during anti-tumor necrosis factor alpha therapy for inflammatory arthritides.. Arthritis Rheum.

[39] Sicotte NL, Voskuhl RR (2001). Onset of multiple sclerosis associated with anti-TNF therapy.. Neurology.

[40] Breedveld FC, Weisman MH, Kavanaugh AF, Cohen SB, Pavelka K (2006). The PREMIER study: A multicenter, randomized, double-blind clinical trial of combination therapy with adalimumab plus methotrexate versus methotrexate alone or adalimumab alone in patients with early, aggressive rheumatoid arthritis who had not had previous methotrexate treatment.. Arthritis Rheum.

[41] (1999). TNF neutralization in MS: results of a randomized, placebo-controlled multicenter study. The Lenercept Multiple Sclerosis Study Group and The University of British Columbia MS/MRI Analysis Group.. Neurology.

[42] Neurath MF, Finotto S (2011). IL-6 signaling in autoimmunity, chronic inflammation and inflammation-associated cancer.. Cytokine Growth Factor Rev.

[43] Sui Y, Potula R, Dhillon N, Pinson D, Li S (2004). Neuronal apoptosis is mediated by CXCL10 overexpression in simian human immunodeficiency virus encephalitis.. Am J Pathol.

[44] Butler GS, Dean RA, Tam EM, Overall CM (2008). Pharmacoproteomics of a metalloproteinase hydroxamate inhibitor in breast cancer cells: dynamics of membrane type 1 matrix metalloproteinase-mediated membrane protein shedding.. Mol Cell Biol.

[45] Xu D, Suenaga N, Edelmann MJ, Fridman R, Muschel RJ (2008). Novel MMP-9 substrates in cancer cells revealed by a label-free quantitative proteomics approach.. Mol Cell Proteomics.

[46] Bai XH, Wang DW, Kong L, Zhang Y, Luan Y (2009). ADAMTS-7, a direct target of PTHrP, adversely regulates endochondral bone growth by associating with and inactivating GEP growth factor.. Mol Cell Biol.

[47] Gossas T, Danielson UH (2006). Characterization of Ca2+ interactions with matrix metallopeptidase-12: implications for matrix metallopeptidase regulation.. Biochem J.

[48] Brown EM, MacLeod RJ (2001). Extracellular calcium sensing and extracellular calcium signaling.. Physiol Rev.

[49] Ramadas RA, Wu L, LeVine AM (2009). Surfactant protein A enhances production of secretory leukoprotease inhibitor and protects it from cleavage by matrix metalloproteinases.. J Immunol.

[50] Song X, Zeng L, Jin W, Thompson J, Mizel DE (1999). Secretory leukocyte protease inhibitor suppresses the inflammation and joint damage of bacterial cell wall-induced arthritis.. J Exp Med.

[51] Mueller AM, Pedre X, Stempfl T, Kleiter I, Couillard-Despres S (2008). Novel role for SLPI in MOG-induced EAE revealed by spinal cord expression analysis.. J Neuroinflammation.

[52] Taggart CC, Cryan SA, Weldon S, Gibbons A, Greene CM (2005). Secretory leucoprotease inhibitor binds to NF-kappaB binding sites in monocytes and inhibits p65 binding.. J Exp Med.

[53] Denis M (1994). Human monocytes/macrophages: NO or no NO?. J Leukoc Biol.

[54] McNeely TB, Dealy M, Dripps DJ, Orenstein JM, Eisenberg SP (1995). Secretory leukocyte protease inhibitor: a human saliva protein exhibiting anti-human immunodeficiency virus 1 activity in vitro.. J Clin Invest.

[55] Lee SC, Liu W, Brosnan CF, Dickson DW (1992). Characterization of human fetal dissociated CNS cultures with an emphasis on microglia.. Lab Invest.

[56] Liu J, Zhao ML, Brosnan CF, Lee SC (1996). Expression of type II nitric oxide synthase in primary human astrocytes and microglia: role of IL-1beta and IL-1 receptor antagonist.. J Immunol.

[57] Suh HS, Zhao ML, Rivieccio M, Choi S, Connolly E (2007). Astrocyte indoleamine 2, 3 dioxygenase (IDO) is induced by the TLR3 ligand poly IC: mechanism of induction and role in anti-viral response.. J Virol.

[58] Krause D, Suh HS, Tarassishin L, Cui QL, Durafourt BA (2011). The tryptophan metabolite 3-hydroxyanthranilic acid plays anti-inflammatory and neuroprotective roles during inflammation: role of hemeoxygenase-1.. Am J Pathol.

[59] Rivieccio MA, Suh HS, Zhao Y, Zhao ML, Chin KC (2006). TLR3 ligation activates an antiviral response in human fetal astrocytes: a role for viperin/cig5.. J Immunol.

